# Association of SMuRFs with cardiovascular risk factors, disease burden, and pharmacological management in Middle Eastern patients with ASCVD and a family history of premature CVD

**DOI:** 10.3389/fcvm.2025.1678767

**Published:** 2025-11-27

**Authors:** Nour Ali Alrida, Zainab Albikawi, Nader Alotaibi, Osama Alkouri, Ahmad Rajeh Saifan, Mohamad Jarrah, Ayman Hammoudeh, Yousef Khader, Awwad Alenezy, Basheer M. Al-Zu’bi, Abdulhafith Alharbi, Fadwa Alhalaiqa, Mohannad E. AbuRuz

**Affiliations:** 1 Clinical Nursing Department, School of Nursing, Yarmouk University, Irbid, Jordan; 2 Faculty of Nursing, Yarmouk University, Irbid, Jordan; 3 College of Nursing, King Saud University, Riyadh, Saudi Arabia; 4 Faculty of Medicine, Jordan University of Science and Technology, Irbid, Jordan; 5 Department of Cardiology, Istishari Hospital, Amman, Jordan; 6 Department of Family and Community Medicine, College of Medicine, Northern Border University, Arar, Saudi Arabia; 7 Allied Medical Professions Department, Al-Balqa Applied University, AS-Salt, Jordan; 8 College of Nursing, University of Hail, Hail, Saudi Arabia; 9 Psychiatric and Mental Health Department, College of Nursing, Qatar University, Al-Dawha, Qatar

**Keywords:** SMuRFs, cardiovascular disease, risk factors, ASCVD, premature CVD

## Abstract

**Introduction:**

Cardiovascular diseases (CVDs) are considered the main reason for death around the world**.** This study investigated the impact of standard modifiable risk factors (SMuRFs) on cardiovascular risk, disease severity, and treatment patterns in Middle Eastern patients who have a history of premature cardiovascular disease, with outcomes including acute coronary syndrome and cerebrovascular accidents.

**Methods:**

We analyzed data from six established cardiovascular registries and the Jordan SMuRF-less cohort study. All datasets were integrated into a single harmonized database to ensure consistency in variable definitions and measurement. The combined dataset included standardized information on participants' demographic and clinical characteristics, cardiovascular risk factors, comorbidities, medication use, and one-year outcomes among individuals categorized as having 0, 1–2, or 3–4 Standard Modifiable Risk Factors (SMuRFs). In total, data from 1,859 participants with atherosclerotic cardiovascular disease (ASCVD) and a family history of early cardiovascular disease were included in the final analysis.

**Results:**

Age varied among the groups. The youngest group (G1) had a mean age of 51.9 ± 12.7 years, while the oldest group (G3) had a mean age of 57.1 ± 10.1 years (*p* < 0.001). CKD and heart failure were more prevalent in the upper SMuRF groups (*p* < 0.001 and *p* < 0.05, respectively). BMI and triglycerides rose with rising SMuRFs (*p* < 0.001), and LDL levels differed between groups (*p* < 0.01). Medication use was highest in G3, especially for statins (94.8% vs. 87.5%), beta blockers (76.0% vs. 66.7%), and oral hypoglycemics (28.6% vs. 2.1%).

**Conclusion:**

The study highlights the significant impact of SMuRFs on cardiovascular health. Older patients with a higher burden of SMuRFs show worse lipid profiles and increased medication use. Given the high prevalence of metabolic syndrome and diabetes in the region, targeted interventions, including lifestyle changes and early pharmacological management, are essential for reducing CVD risk in Middle Eastern populations.

**Clinical Trial Registration:**

ClinicalTrials.gov, NCT06199869.

## Introduction

Cardiovascular diseases (CVDs) remain the foremost cause of global mortality, with atherosclerotic cardiovascular disease (ASCVD) identified as the leading contributor to these deaths (1). In the Middle East, the burden of ASCVD has heightened in recent years due to a complex interplay of genetic susceptibility, rapid urbanization, dietary transitions, and sedentary behaviors (2). This shift has made the region a focal point for preventive cardiology, particularly concerning Standard Modifiable Risk Factors (SMuRFs), a term encompassing well-established and preventable clinical factors such as smoking, hypertension, diabetes mellitus, and dyslipidemia, each of which significantly heightens the risk for ASCVD (3).

SMuRFs often cluster together, creating a collaborative and harmful effect on cardiovascular health that exacerbates vascular damage and accelerates disease progression. Their cumulative presence increases the likelihood of adverse outcomes, including myocardial infarction, cerebrovascular accidents (CVA), and heart failure (4, 5). In regions like the Middle East, where culturally rooted barriers and inconsistent access to healthcare prevail, comprehensive evaluation and management of SMuRFs remain essential yet underdeveloped. Age further complicates ASCVD management, serving as a non-modifiable risk factor that amplifies vulnerability to comorbidities such as chronic kidney disease (CKD) and heart failure (6, 7). With aging populations in the region, a growing number of patients present with multiple intersecting risk factors, demanding a patient-centered, stratified approach to treatment rather than a one-size-fits-all model.

Recent Middle Eastern data indicate that a significant proportion of ASCVD patients have multiple SMuRFs, with associated elevations in LDL and triglycerides and reductions in HDL (4, 8). Moreover, the presence of ≥3 SMuRFs is linked to a higher incidence of recurrent cardiovascular events, highlighting the need for early identification and targeted intervention (9).

Socioeconomic transformation and cultural factors in the Middle East have also influenced the patterns of CVD risk. Economic development and changing dietary habits have contributed to rising rates of obesity and metabolic syndrome, particularly among younger populations (10). The family history of premature CVD, a non-modifiable but clinically significant marker, further compounds the risk in this population. Premature CVD refers to cardiovascular events such as heart attacks or strokes occurring before the age of 55 in men and 65 in women (11). Elevated body mass index (BMI) has emerged as a key concern, with global studies confirming its strong association with ASCVD when coupled with multiple SMuRFs (12, 13).

Pharmacologic management plays a central role in secondary prevention. Medications such as statins, beta-blockers, antiplatelet agents, and glucose-lowering therapies are cornerstones of modern ASCVD care (2). Evidence suggests that patients with a greater number of SMuRFs are more likely to receive these therapies (2, 14). However, variability in prescription practices and adherence remains a challenge, often influenced by comorbid burden, socioeconomic disparities, and clinical inertia (15, 16).

Despite a growing body of literature, important gaps persist. The precise impact of SMuRF clustering on individual cardiovascular endpoints, particularly in populations with a family history of premature CVD, has not been well quantified (9, 17). Moreover, there is limited data on how SMuRF burden influences pharmacological decision-making in real-world settings in the Middle East. The interaction between cultural context, risk factor clustering, and therapeutic outcomes remains poorly understood. This study aimed to investigate the association between SMuRFs and cardiovascular risk, disease burden, and treatment patterns among Middle Eastern patients with ASCVD and a family history of premature CVD. It seeks to evaluate the prevalence and clustering of SMuRFs, explore associations with comorbidities such as CKD and heart failure, and assess trends in pharmacological management. Findings from this study will contribute to a more refined understanding of ASCVD risk stratification and to the development of culturally contextualized prevention and treatment strategies in the Middle East. By highlighting the interplay between SMuRF burden, comorbid conditions, and therapeutic patterns, the study aims to support the development of tailored clinical guidelines and inform public health interventions to reduce ASCVD-related morbidity and mortality in this high-risk population.

## Materials and methods

### Study design

This study utilized a harmonized cohort design that integrated data from multiple established cardiovascular registries and one ongoing national cohort in Jordan. Specifically, the analysis combined prospectively collected data from the Jordan SMuRF-less study (ClinicalTrials.gov identifier: Record History|ver. 1: 2024-01-09|NCT06199869|ClinicalTrials.gov), conducted between January 10 and August 20, 2024, with retrospectively extracted data from six pre-existing cardiovascular registries across the Middle East. All datasets contributed standardized patient-level information on demographics, cardiovascular risk factors, comorbidities, medications, and one-year clinical outcomes.

To ensure consistency and comparability across sources, variables from each registry were reviewed, aligned, and harmonized according to a unified data dictionary. The research team resolved duplicate variables and inconsistent definitions through consensus. Thus, although the registries themselves varied in collection timelines, all analyses in the present study were conducted retrospectively using harmonized, de-identified datasets. The included registries were: the First Jordan Percutaneous Coronary Intervention Registry (NCT01841346) (18). The Study of Novel and Classical Risk Factors in Young Middle Eastern Women with ASCVD (NCT04975503) (19). The Surviving a Decade or More after Coronary Revascularization Study (NCT03491722) (20). The Jordan Atrial Fibrillation Study (NCT03917992) (21). The Jordan Atrial Fibrillation Study (duplicate listing of NCT03917992) (22). The Jordan COVID-19 Pandemic Acute Cardiovascular Events Study (NCT04368637) (2, 23). All data were collected by trained research coordinators using standardized case report forms. The recorded variables included demographic and anthropometric data, medical history, conventional and emerging cardiovascular risk factors (including both modifiable and non-modifiable factors), comorbidities, pharmacologic treatments used for secondary prevention, and one-year survival outcomes following the initial cardiovascular event.

This review of cardiovascular registries from Jordan and the Middle East (see Table 1) was used to contextualize population characteristics and disease patterns. These registries offer regionally relevant insights into cardiovascular disease across diverse demographics and healthcare settings. Their findings are broadly applicable to similar Middle Eastern contexts and support comparisons with Western populations, enhancing understanding of regional differences in disease outcomes and management. This adds depth to the current study's methodological framework and strengthens the generalizability of its findings.

**Table 1:** Overview of cardiovascular-related registries involving Jordan and the Middle East.

Registry Name	Registry ID	Objective	Timeframe	Inclusion Criteria	Exclusion Criteria	Patient Characteristics	Cities Involved
Jordan SMuRF-less Study	NCT06199869	Assess risk factors for ASCVD in Jordanian adults beyond traditional SMuRFs	Jan 10, 2024—Aug 20, 2024	Adults ≥18 years with ASCVD	Congenital heart disease, pregnancy/lactation, inability to consent	Jordanian adults with ASCVD, focusing on less common risk factors	3 community hospitals, 6 tertiary centers across Jordan (MOH, university, private)
First Jordan PCI Registry	NCT01841346	Track outcomes, complications, and effectiveness of PCI in Jordan	Ongoing	Adults ≥18 years undergoing PCI with confirmed CAD	Contraindications to PCI, refusal to consent, interfering comorbidities	Patients undergoing PCI (elective/emergency), various ages	Hospitals across Jordan performing PCI
ASCVD Risk Factors in Young Women	NCT04975503	Identify novel and classical risk factors for ASCVD in young ME women.	Long-term project	Females aged 18–45 with or at risk of ASCVD	Pregnancy, prior CABG/major CV events, significant chronic diseases	Young Middle Eastern women	Urban centers in the Middle East (unspecified)
Survival Post-Coronary Revascularization	NCT03491722	Analyze long-term outcomes after coronary revascularization	Long-term survivors >10 years	Adults surviving ≥10 years post-PCI or CABG	Surgeries <10 years, terminal illnesses	Middle Eastern long-term CAD survivors	Various Middle Eastern hospitals including Jordan
Jordan Atrial Fibrillation Study	NCT03917992	Evaluate prevalence, management, and outcomes of AF in Jordan.	Ongoing	Adults ≥18 years with AF	Non-cardiac arrhythmias, pregnancy, contraindications to anticoagulants	AF patients (new and existing cases)	Jordanian hospitals specializing in cardiology and arrhythmia
Statin Eligibility in AMI Patients	NCT03485742	Assess statin eligibility among Middle Eastern AMI patients	Not specified	Adults ≥18 years with AMI, eligible for statins	Contraindications to statins, prior adverse reactions	Middle Eastern AMI patients	Hospitals with high AMI case volume in Middle East
Jordan COVID-19 Acute Cardiovascular Events Study	NCT04368637	Understand COVID-19′s impact on acute CV events in Jordan	During COVID-19 pandemic	Adults ≥18 years with acute CV events during pandemic	No CV events during pandemic, confounding conditions.	Acute CV patients during COVID-19	Jordanian hospitals treating COVID-19 and cardiovascular emergencies

This table summarizes key characteristics of seven cardiovascular-related registries that include Jordanian or Middle Eastern populations. It outlines each registry's name, ClinicalTrials.gov identifier, primary objective, study timeframe, inclusion and exclusion criteria, patient characteristics, and the geographic distribution of participating hospitals or centers. These registries represent a mix of prospective and retrospective studies addressing various cardiovascular conditions such as ASCVD, atrial fibrillation, coronary revascularization outcomes, and cardiovascular complications during the COVID-19 pandemic.

### Study setting

The study was conducted across multiple medical centers in Jordan and incorporated data from regional cardiovascular registries in the Middle East. For the Jordan SMuRF-less study, participants were recruited from 9 medical centers across Jordan, ensuring diversity in healthcare access and patient backgrounds. These centers included: three community hospitals, three Ministry of Health hospitals, two university-affiliated hospitals, one private academic hospital. This comprehensive coverage included institutions from both public and private sectors, as well as academic and community-based settings, ensuring a representative sample of the Jordanian population. The *post hoc* registry data provided additional cases from across the Middle East, reflecting a multinational scope of ASCVD presentation and management, and strengthening the regional relevance and generalizability of the study findings.

### Inclusion criteria and definition of exposures

The study involved patients with atherosclerotic cardiovascular disease (ASCVD), including coronary artery disease (CAD), stroke, carotid artery disease, and peripheral artery disease. Within the CAD population, distinct subgroups were established. They included patients with acute coronary syndrome (ACS), including ST-segment elevation myocardial infarction (STEMI) and non-ST elevation ACS, and those who presented with chronic stable angina (CSA) and CAD documented by coronary computed tomography angiography (CCTA). They were divided into three groups based on the number of standard modifiable risk factors (SMuRFs): participants with no SMuRFs, one or two SMuRFs, and three or four SMuRFs.

### Definitions of SMuRFs

The SMuRFs were defined as binary variables based on established criteria. The diagnostic criteria for hypertension (HTN), type 2 diabetes (T2D), elevated serum LDL-C levels, and cigarette smoking were consistent with those used in previous studies (2, 24–28). HTN was diagnosed if a healthcare provider had a prior diagnosis, used antihypertensive medications, or a new diagnosis during hospitalization with repeated measurements of systolic blood pressure ≥140 mm Hg and/or diastolic blood pressure ≥90 mm Hg. Type 2 diabetes was defined by a prior diagnosis, use of glucose-lowering drugs, or a hemoglobin A1c level ≥6.5%. Dyslipidemia was determined by a previous diagnosis from a healthcare provider, use of lipid-lowering medications, or elevated serum LDL-C levels above recommended target levels. A participant was classified as a current smoker if they had smoked regularly within the past year before enrollment.

### Definitions of other traditional risk factors

The evaluation also contained a traditional risk factor: family history of early development of CVD, which was considered a cardiovascular event in a first-degree male relative below the age of 55 or a female relative before the age of 65.

### Ethical consideration

This observational study adhered to the ethical principles outlined in the Declaration of Helsinki. For the prospective cohort data, ethical approval, including Institutional Review Board approval, was obtained from all participating institutions, including approval from the Institutional Review Board/Independent Ethics Committee at Istishari Hospital in Amman, Jordan (ID number: IRBIG/AH/11/01/24), Date (11 January 2024). All participants signed a written informed consent form. This study has been registered at ClinicalTrials.gov under the reference (Record History|ver. 1: 2024-01-09|NCT06199869|ClinicalTrials.gov). For the retrospective cohort design, the data analyzed in this study were drawn from a database of 5,540 patients who had participated in previously published cardiovascular studies, all of which were registered on ClinicalTrials.gov. Informed consent had been obtained at the time of enrollment in those studies, as reported in the original publications. For the current retrospective analysis, which used pooled and fully de-identified data, the IRB waived the requirement for new consent. This waiver was granted due to the lack of identifiable patient information and the extended time since the original data collection, which made follow-up or re-contact with participants impractical.

### Statistical analysis

Statistical analyses were carried out using SPSS version 26. To compare the three groups, SMuRFS-Less (G1), one to two SMuRFS (G2), and three to four SMuRFS (G3), chi-square tests were used for categorical variables, and one-way analysis of variance (ANOVA) with Bonferroni *post hoc* tests was used for continuous variables. A *p*-value of less than 0.05 was considered statistically significant for all analyses.

## Results

Table 2 presents the sociodemographic and clinical characteristics of the total study sample (*N* = 1859), stratified by SMuRFs group: SMuRF-less (Group 1, *n* = 48), one to two SMuRFs (Group 2, *n* = 903), and three to four SMuRFs (Group 3, *n* = 908). There was a statistically significant difference in age across the three groups (*p* < 0.001). The SMuRF-less group was the youngest, with a mean age of 51.9 ± 12.7 years, while participants with three to four SMuRFs were the oldest, with a mean age of 57.1 ± 10.1 years. The gender distribution did not differ significantly across the groups (*p* = 0.242).

**Table 2:** Sociodemographic and clinical characteristics of the study sample by SMuRF counts (*N* = 1859).

Variable	Total sample (*N* = 1,859)	(G1): SMuRFS-Less (*n* = 48)	(G2) one to two SMuRFS (*n* = 903)	(G3) three to four SMuRFS (908)	*F* test or *X^2^, p* value	Effect size	95% CI
Age	55.5 ± 10.9	51.9 ± 12.7	54.0 ± 11.3	57.1 ± 10.1	<0.001**	*η*^2^ = 0.04	54.8–56.1
Gender							
Male	1,384 (74.4)	31 (64.6)	683 (75.6)	670 (73.8)	0.242	Cramer's V = 0.06	Male: 74.4% (72.3–76.4)
Female	475 (25.6)	17 (35.4)	220 (24.4)	238 (26.2)	0.242		
Hypertension	1,128 (60.7)	0 (0)	327 (36.2)	801 (88.2)	<0.001**	Cramer's V = 0.62	58.3–63.1
Diabetes mellitus	1,002 (53.9)	0 (0)	246 (27.2)	756 (83.3)	<0.001**	Cramer's V = 0.59	51.5–56.3
Dyslipidemia	1,436 (77.2)	0 (0)	575 (63.7)	861 (94.8)	<0.001**	Cramer's V = 0.53	75.2–79.1
Smoking	940 (50.6)	0 (0)	392 (43.4)	548 (60.4)	<0.001**	Cramer's V = 0.21	48.3–52.8
CKD	79 (4.2)	0 (0)	23 (2.5)	56 (6.2)	<0.001**	Cramer's V = 0.09	3.3–5.1
Heart Failure	233 (12.5)	4 (8.3)	94 (10.4)	135 (14.9)	0.018*	Cramer's V = 0.08	11.1–13.9
Diagnosis *n* (%)							
ACS	1,533 (82.5)	40 (83.3)	750 (83.1)	743 (81.8)	0.376	Cramer's V = 0.02	80.9–84.1
CVA	98 (5.3)	5 (10.4)	46 (5.1)	47 (5.2)	0.376	Cramer's V = 0.05	4.3–6.3
Chronic stable angina	228 (12.3)	3 (6.3)	107 (11.8)	118 (13.0)	0.376	Cramer's V = 0.03	10.8–13.8
BMI (kg/m^2^)	28.7 ± 4.8	27.1 ± 3.6	28.3 ± 4.7	29.3 ± 5.0	<0.001**	η^2^ = 0.03	28.3–29.1
LDL (mg/dL)	117.6 ± 48.5	97.3 ± 28.5	121.4 ± 48.8	114.5 ± 48.5	0.007*	η^2^ = 0.01	115.3–119.9
Total cholesterol (mg/dL)	185.2 ± 56.1	169.8 ± 43.4	187.2 ± 55.6	183.7 ± 56.8	0.124	η^2^ = 0.008	182.1–188.3
Triglycerides (mg/dL)	127.0 ± 163.2	54.7 ± 97.9	113.8 ± 136.9	144.0 ± 186.2	<0.001**	η^2^ = 0.02	116.1–137.9
HDL (mg/dL)	38.6 ± 14.6	42.2 ± 8.8	39.2 ± 11.6	37.9 ± 21.8	0.087	η^2^ = 0.005	37.5–39.7

Values are presented as mean ± SD or *n* (%). Statistical significance was assessed using *F* test or Chi-square test.

ACS, acute coronary syndrome; CVA, cerebrovascular accident; CKD, chronic kidney disease; BMI, body mass index; SMuRF, standard modifiable risk factor.

* Significant at *p* < 0.05; **Significant at *p* < 0.001.

The prevalence of chronic comorbidities varied significantly by SMuRF status. Specifically, the history of chronic kidney disease (CKD) and heart failure increased significantly with the number of SMuRFs (*p* < 0.001 and *p* = 0.018, respectively). The proportion of patients with a history of hypertension, diabetes mellitus, dyslipidemia, and smoking rose progressively across SMuRF groups (all *p* < 0.001), reflecting expected clustering patterns of these modifiable risk factors. In contrast, the type of cardiovascular presentation, including acute coronary syndrome (ACS), cerebrovascular accident (CVA), and chronic stable angina, did not significantly differ among the groups (*p* = 0.376). Body Mass Index (BMI) was significantly higher in participants with a greater number of SMuRFs (*p* < 0.001), increasing from a mean of 27.1 ± 3.6 kg/m^2^ in Group 1–29.3 ± 5.0 kg/m^2^ in Group 3.

Regarding lipid parameters, low-density lipoprotein (LDL) levels varied significantly among the groups (*p* = 0.007), with the highest mean LDL observed in Group 2 (121.4 ± 48.8 mg/dL). Triglyceride levels were different across groups (*p* < 0.001), increasing with the number of SMuRFs; the lowest levels were recorded in the SMuRF-less group (54.7 ± 97.9 mg/dL), and the highest in Group 3 (144.0 ± 186.2 mg/dL). No significant differences were found in total cholesterol (*p* = 0.124) or high-density lipoprotein (HDL) levels (*p* = 0.087) between groups.

Table 3 outlines the use of cardiovascular-related medications across SMuRF groups. Several pharmacologic therapies demonstrated significant variations in prescription patterns by SMuRF burden. The use of statins and aspirin was significantly more prevalent among patients in Group 3 (94.8% for both) compared to those in Group 1 (87.5%) (*p* = 0.032 for both). A similar trend was observed in beta-blocker usage, with 76.0% of Group 3 patients receiving these medications compared to 66.7% in the SMuRF-less group (*p* < 0.001). The administration of oral hypoglycemic agents differed by group, with usage highest among those with three to four SMuRFs (28.6%) and lowest in the SMuRF-less group (2.1%) (*p* < 0.001), consistent with the high prevalence of diabetes in this group. No statistically significant differences were observed across groups in the use of clopidogrel (*p* = 0.181), other P2Y12 inhibitors (*p* = 0.211), or dual antiplatelet therapy (*p* = 0.198).

**Table 3:** Comparisons of medication use among the three groups (*N* = 1,859).

Medication used	Total sample (*N* = 1,859)	(G1): SMuRFS-Less (*n* = 48)	(G2) one to two SMuRFS (*n* = 903)	(G3) three to four SMuRFS (908)	*X^2^, p* value	Effect size	95% CI
Statins	1,741 (93.7)	42 (87.5)	838 (92.8)	861 (94.8)	0.032*	Cramer's V = 0.11	92.5–94.8
Aspirin	1,740 (93.6)	42 (87.5)	837 (92.7)	861 (94.8)	0.032*	Cramer's V = 0.11	92.4–94.7
Clopidogrel	1,143 (61.5)	33 (68.8)	544 (60.2)	566 (62.3)	0.181	Cramer's V = 0.05	59.4–63.5
P2Y12 inhibitors	293 (15.8)	4 (8.3)	141 (15.6)	148 (16.3)	0.211	Cramer's V = 0.04	14.2–17.3
Dual antiplatelet therapy	1,377 (74.1)	35 (72.9)	651 (72.1)	691 (76.1)	0.198	Cramer's V = 0.04	72.3–75.8
Beta blockers	1,325 (71.3)	32 (66.7)	603 (66.8)	690 (76.0)	<0.001**	Cramer's V = 0.12	69.9–72.6
Oral hypoglycemic agents	341 (18.3)	1 (2.1)	80 (8.9)	260 (28.6)	<0.001**	Cramer's V = 0.36	16.6–20.0

Comparison of medication use among the three groups based on the number of SMuRFS (*N* = 1,859). Values are presented as number (percentage). Statistical comparisons were made using the Chi-square test.

* Significant at <0.05.

** Significant at <0.001.

## Discussion

The current study examined the associations between SMuRFs and cardiovascular risk, disease burden, and treatment patterns among Middle Eastern patients with ASCVD and a family history of premature CVD, by evaluating the prevalence and clustering of SMuRFs, exploring associations with comorbidities such as CKD and heart failure, and assessing trends in pharmacological management. The results demonstrate associations between sociodemographic characteristics, such as age, gender, and health behaviors, and the prevalence of SMuRFs, which may influence health outcomes.

Age was a key variable; patients with three to four SMuRFS tended to be considerably older than patients without risk factors. This observation is consistent with evidence that has shown an increased prevalence of cardiovascular risk factors in direct proportion to increased age, in that elderly populations have greater rates of dyslipidemia, hypertension, and diabetes (26, 29–31). Furthermore, this cohort exhibited higher frequencies of hypertension, diabetes mellitus, dyslipidemia, and smoking behaviors, reflecting a cumulative risk burden potentially associated with adverse cardiovascular outcomes (28, 32, 33).

The analysis of body mass index (BMI) across patient groups revealed important trends. Higher BMI values were associated with greater clustering of SMuRFs, particularly among individuals in Group 3, whose mean BMI was in the obese range. This supports evidence suggesting a link between obesity and increased cardiometabolic risk and chronic conditions such as heart failure (34, 35). Notably, obesity appears to contribute to the burden of heart failure in the Middle Eastern population. For example, an Egyptian study reported obesity prevalence among patients hospitalized for decompensated heart failure at 39.7% of men and 61.2% of women (36, 37). Heart failure has been observed at a younger age in Middle Eastern populations, up to a decade earlier than in Western cohorts, likely due to earlier and more frequent exposure to risk factors such as diabetes mellitus, obesity, and hypertension (38, 39). Regional epidemiological data also indicate a high prevalence of metabolic syndrome, where obesity plays a central role; for instance, a study in the UAE reported a 37.4% prevalence, driven mainly by elevated BMI (40). These findings suggest the importance of targeting obesity within the broader SMuRF framework to reduce early-onset ASCVD and related comorbidities.

Examination of lipid profiles revealed that patients with three to four SMuRFs tended to have higher triglyceride levels, along with increased LDL and reduced HDL, compared to other groups (41, 42). The rise in triglycerides was particularly pronounced in Group 3, highlighting a triglyceride-specific association with multiple SMuRF clustering. These findings underscore the importance of monitoring triglyceride levels as a distinct marker of cardiometabolic risk. Regional studies indicate that hypertriglyceridemia and dyslipidemia are common in Gulf Cooperation Council (GCC) countries, with prevalence ranging between 26% and 50.7% among men and 20.9% and 57.2% among women, increasing with age (43). These observations highlight associations between triglyceride elevations and SMuRF clustering in Middle Eastern populations.

The prevalence of CKD and heart failure was higher in patients with three to four SMuRFs, consistent with additive comorbidity patterns; these findings highlight the need for careful monitoring in patients with multiple cardiovascular risk factors (37, 44). The heightened prevalence of CKD is noted because renal dysfunction can both result from and amplify cardiovascular disease through pathways such as hypertension-related nephropathy and dyslipidemia-related endothelial damage. These findings highlight the importance of monitoring patients with multiple cardiovascular risk factors (17, 45).

Family history should not be underestimated as an independent determinant of cardiovascular risk. Evidence suggests that patients with a family history of CVD have higher rates of traditional cardiovascular risk factors (46, 47). Smoking prevalence was also higher among patients with more SMuRFs, consistent with associations between smoking and cardiovascular risk (43). Regional smoking rates vary from 25% to 52% among men, with increased waterpipe use further contributing to risk (48). Given the well-established association between smoking and accelerated atherosclerosis, as well as its contribution to hypertension and dyslipidemia, these results highlight the critical need for focused tobacco control policies and smoking cessation programs (49) These results highlight the need for targeted public health initiatives, such as tobacco control and cessation programs (50).

In terms of pharmacologic management, statins and aspirin were widely used, with the highest use observed in patients with three or four SMuRFs (G3). This trend aligns with current recommendations for lipid-lowering therapy and antiplatelet use in secondary ASCVD prevention (50). Given that, dyslipidemia was much more prevalent in G3 patients, the use of statins follows guidelines issued by the European Society of Cardiology (ESC) and American College of Cardiology (ACC), which prescribe intense lipid control in vulnerable patient populations (51). Similarly, aspirin use was highest in G3 patients, perhaps as a reflection of a higher prevalence of conditions like stroke and myocardial infarction, in whom there is proven benefit for recurrent ischaemic events (52). Conversely, the slightly lower use in SMuRFS-less (G1) patients (87.5%) may reflect a lower felt need for primary prevention in the absence of traditional risk factors.

Beta-blocker prescription was more frequent in Group 3 (76.0%) compared to G1 and G2, reflecting associations with a higher comorbidity burden and aligning with clinical guidelines for beta-blockers in the management of hypertension, prior myocardial infarction, and heart failure (53). Because of their role, beta-blockers play a central role in reducing myocardial oxygen demand, treating hypertension, and preventing arrhythmias, each of which is more prevalent in high-SMuRFS, reflecting their increased comorbidity burden. The most prominent medication use was in oral hypoglycemics, and they were far more common in G3 compared to G1 and G2.

Given the Middle Eastern prevalence rates among the highest worldwide, this trend fits the significant burden of type 2 diabetes mellitus (T2DM). The International Diabetes Federation (IDF) reports that diabetes rates vary across Gulf Cooperation Council (GCC) nations from 8% to 22%. Among men, some nations show especially higher frequency: Bahrain (33.6%), Saudi Arabia (29.1%), United Arab Emirates (25.83%), and Kuwait (25.4%) (54, 55). The close link between T2DM and ASCVD is established as T2DM is known to accelerate atherosclerosis and predispose to cardiovascular events, irrespective of other risk factors (56). As a direct implication of increased prevalence in the region due to increased sedentariness, calorie-surplus diet, and genetic predisposition, increased use of oral hypoglycemic medication in multiple cases in the SMuRF represents an increased need for strict glycemic and cardiovascular risk control in the population in the Middle East (57). In response to this challenge, sodium-glucose cotransporter 2 (SGLT2) inhibitors and glucagon-like peptide-1 (GLP-1) receptor agonists are recommended for managing cardiovascular risk in patients with diabetic ASCVD (58).

In summary, this study demonstrates associations between higher SMuRF counts and a greater cardiometabolic burden, increased prevalence of comorbidities, and more intensive pharmacologic management among Middle Eastern patients with ASCVD and a family history of premature CVD. Patients with three to four SMuRFs tended to exhibit higher age, BMI, triglycerides, and LDL levels, established markers of cardiovascular risk. These findings indicate an association between higher SMuRF counts and increased prevalence of comorbidities, highlighting the importance of closer clinical monitoring in patients with multiple coexisting SMuRFs.

The findings provide region-specific evidence on how SMuRF clustering correlates with disease burden, comorbidity patterns, and treatment practices, emphasizing that patients with multiple risk factors may require comprehensive, individualized care. Clinicians may benefit from adopting risk-stratification frameworks that incorporate SMuRF counts to prioritize screening, preventive strategies, and early interventions. Furthermore, addressing behavioral and lifestyle risk factors, such as obesity, smoking, and poor glycemic control, through culturally sensitive public health strategies and patient education may help reduce long-term cardiovascular morbidity.

Overall, a multidimensional approach integrating early identification, personalized management, pharmacologic optimization, and lifestyle interventions may be important for mitigating cardiovascular risk in Middle Eastern populations with multiple SMuRFs. Future research should examine longitudinal outcomes, intervention effectiveness, and the potential impact of modifying SMuRF clusters on cardiovascular event rates.

Nevertheless, this study has important limitations. First, the observational cohort design limits causal inference; associations observed cannot confirm direct cause-and-effect relationships. Second, the very small SMuRF-less group (*n* = 48) reduces statistical power, making comparisons underpowered and potentially less reliable. Third, although data were drawn from six cardiovascular registries, potential selection bias and heterogeneity across registries may have influenced results; future studies could mitigate this by standardizing data collection and performing sensitivity analyses. Fourth, generalizability beyond the Middle East is limited. Fifth, behavioral variables, such as diet, exercise, and medication adherence, were not assessed. Finally, although family history of premature CVD was included, it was not analyzed as an effect modifier, potentially overlooking its influence.

Despite these limitations, the study provides valuable region-specific evidence by systematically evaluating the intersection of SMuRFs, comorbidities, and pharmacological interventions in a high-risk Middle Eastern population. Future longitudinal research should incorporate broader psychosocial, behavioral, and family history variables, as well as follow-up beyond one year, to strengthen risk prediction models and optimize cardiovascular care delivery.

## Conclusion

This study reinforces the critical need for early, targeted, and culturally tailored cardiovascular risk reduction strategies in the Middle East, particularly among individuals with a family history of premature CVD. The clustering of modifiable risk factors highlights the importance of integrated prevention and management approaches that address not only individual behaviors but also broader healthcare system challenges. Strengthening screening programs, enhancing public awareness, and optimizing treatment protocols may help mitigate the escalating burden of ASCVD in the region and improve long-term outcomes for high-risk populations.

## Data Availability

The original contributions presented in the study are included in the article/Supplementary Material, further inquiries can be directed to the corresponding authors.
